# Curriculum Mapping of the Master’s Program in Pharmacy in Slovenia with the PHAR-QA Competency Framework

**DOI:** 10.3390/pharmacy5020024

**Published:** 2017-05-02

**Authors:** Tanja Gmeiner, Nejc Horvat, Mitja Kos, Aleš Obreza, Tomaž Vovk, Iztok Grabnar, Borut Božič

**Affiliations:** Faculty of Pharmacy, University of Ljubljana, Askerčeva 7, 1000 Ljubljana, Slovenia; Tanja.Gmeiner@ffa.uni-lj.si (T.G.); Nejc.Horvat@ffa.uni-lj.si (N.H.); Mitja.Kos@ffa.uni-lj.si (M.K.); Ales.Obreza@ffa.uni-lj.si (A.O.); Tomaz.Vovk@ffa.uni-lj.si (T.V.); Iztok.Grabnar@ffa.uni-lj.si (I.G.)

**Keywords:** pharmacy education, competences, curriculum mapping, community pharmacy, industrial pharmacy, clinical pharmacy, Delphi study, quality assurance, European framework

## Abstract

This article presents the results of mapping the Slovenian pharmacy curriculum to evaluate the adequacy of the recently developed and validated European Pharmacy Competences Framework (EPCF). The mapping was carried out and evaluated progressively by seven members of the teaching staff at the University of Ljubljana’s Faculty of Pharmacy. Consensus was achieved by using a two-round modified Delphi technique to evaluate the coverage of competences in the current curriculum. The preliminary results of the curriculum mapping showed that all of the competences as defined by the EPCF are covered in Ljubljana’s academic program. However, because most EPCF competences cover healthcare-oriented pharmacy practice, a lack of competences was observed for the drug development and production perspectives. Both of these perspectives are important because a pharmacist is (or should be) responsible for the entire process, from the development and production of medicines to pharmaceutical care in contact with patients. Nevertheless, Ljubljana’s graduates are employed in both of these pharmaceutical professions in comparable proportions. The Delphi study revealed that the majority of differences in scoring arise from different perspectives on the pharmacy profession (e.g., community, hospital, industrial, etc.). Nevertheless, it can be concluded that curriculum mapping using the EPCF is very useful for evaluating and recognizing weak and strong points of the curriculum. However, the competences of the framework should address various fields of the pharmacist’s profession in a more balanced way.

## 1. Introduction

Traditional universities structured programs with a defined number of courses, exams, and contact hours. It was up to the teachers to know what students needed in order to graduate from the university. The system was rather clear and worked smoothly. The majority of older pharmacists received their degrees through education structured in this way, and the pharmacy profession developed well, even excellently. Three independent factors resulted in a need to change this mindset in order to introduce competence-oriented curricula: (a) a significantly greater amount of information (not necessarily knowledge), (b) a shorter half-life of research-based knowledge, and (c) an increasing number of universities due to drastic changes in the expectations of the general population. Namely, only 2% of the population was expected to participate in higher education in the 19th century, compared to the European trend of the 21st century, in which 40% of the population is expected to participate in higher education. The change is not an issue of quantity alone, but also a question of quality. To meet the needs and expectations of society, curricula need to be reoriented from a structured mode to a competence-oriented mode [1,2].

Pharmacy education has deep roots in Slovenia. The principles of quality work in the pharmaceutical profession were introduced as early as in the 17th and 18th centuries. In 1710, a Pharmaceutical code was introduced for the Duchy of Carniola. Under the Illyrian Provinces at the beginning of the 19th century, pharmacy was taught through *materia medica* and pharmaceutical chemistry as the main subjects at the Central school in Ljubljana. Competences in pharmaceutical technology were built through traineeship at community or hospital pharmacies. University teaching of pharmacy was established in Ljubljana in the mid-20th century, with the first attempts in 1946 and 1955 as a two-year program, and starting in 1960 as a complete eight-semester program [3]. The development of undergraduate pharmacy education including clinical chemistry was based on the connection between research and practical applications in all fields of the pharmaceutical profession and science. The program was revamped several times, and it was extended to a four-and-a-half-year program in the mid-1990s. To show the integrity of the competences obtained, the curricula included awarding a diploma for individual student research work from the very beginning. After receiving their degrees, the graduates were employed as healthcare professionals (at community pharmacies, hospital pharmacies, and medical laboratories), researchers (in the public or private sector), in the pharmaceutical industry (in all four sectors: research and development, production, quality assurance, and marketing and sales), as teachers (at high schools and universities), or as professionals in pharmaceutical legislation. For employment, graduates needed to complete a probationary period and pass the final state exam. Several minor changes in the probation period based on future employers’ needs were introduced before the program was harmonized according to European directives in 2004, when Slovenia entered the EU. Six months of traineeship in a pharmacy was included in the curriculum in the last semester of the five-year program [4]. Finally, the program was revamped and improved as a part of the Bologna process to a 10-semester uniform masters program: eight semesters of lectures, seminars, lab work, and other activities, one semester (6 months) of traineeship in pharmacies, and one semester of individual research work for the master’s thesis. The state exam for pharmacists as healthcare professionals was integrated into the last semester, and was completed with a public defense of the master’s thesis. The program was accredited by the National Agency for Quality in Higher Education in 2007 and was reaccredited in 2015 [5].

Several stakeholders were involved in the process of reform and accreditation through roundtables, workshops, meetings, written opinions, and other means. These included teachers from the university faculties involved (pharmacy, chemistry, medicine, mathematics, and physics), students (through the student counsel and the pharmacy students association), graduates, professional societies and chambers (the Slovenian Pharmaceutical Society, the Slovenian Chamber of Pharmacies, and the Slovenian Chamber of Laboratory Medicine), potential employers such as directors of community and hospital pharmacies, generic and innovative industry, and regulators (the Ministry of Health, and the Public Agency for Medicinal Products and Medical Devices). With this approach, we addressed recommendations by High Level Group on the Modernization of Higher Education, published in 2013 [1]. Namely, the program provides competences for employment in community pharmacies, hospital pharmacies, the pharmaceutical industry, medical laboratories, research laboratories, legislation, and education [6]. In some areas, an additional three or four years of specialization (as training) is necessary for special areas of the pharmacy profession, such as specializations in clinical pharmacy, medical design, medical testing, clinical chemistry, and radiopharmacy. Doctoral study is open after a degree in several fields, such as pharmacy, clinical chemistry and laboratory medicine, toxicology, biochemistry and molecular biology, and genetics [7].

The master’s program in pharmacy was designed for the first-day-of-job-pharmacist; that is, for novices or beginners with limited experience [8] to be able to work autonomously. During the education process, competences are built from lower to higher levels, and therefore horizontal and vertical course linkages are very important. The primary objective of the Faculty of Pharmacy is to develop scientifically and professionally qualified, high-quality graduates familiar with ethical principles that autonomously carry out demanding tasks in community and hospital pharmacies, in all fields of the pharmaceutical industry, in clinical laboratories and laboratory medicine, laboratories for drug control and analysis, research institutions, educational organizations, state bodies, and wherever the work and presence of a pharmacist is required to increase health safety [9]. The faculty’s commitment to quality teaching and research has been shown through many activities, including participation in projects initiated by EAFP [10], such as Pharmacy Education in Europe (Pharmine) and Quality Assurance in European Pharmacy Education and Training (PHAR-QA).

The European Commission has funded the international project PHAR-QA [11] to produce a consensual, harmonized framework of competences for pharmacy practice across Europe. This framework is intended to be used as a base for a QA system for evaluating university pharmacy education and training at the institutional, national, and/or European levels [12]. The second round of the PHAR-QA survey of competences for pharmacy practice in Europe was completed in 2016 [13].

The aim of this study was to evaluate the usefulness of the framework developed for pharmaceutical competences as a tool for mapping the master’s pharmacy curricula by matching the existing curriculum of the master’s program in pharmacy in Slovenia to the framework.

## 2. Materials and Methods

A team of seven members of the teaching staff in the integrated master’s program in pharmacy [6] at the University of Ljubljana’s Faculty of Pharmacy was involved in curriculum mapping. Two members of the team have previously been involved in the PHAR-QA project [11]; three members are responsible for coordinating the master’s program, international student exchange, and traineeship as part of undergraduate study; and four members of the team are also members of the faculty management. The mapping was carried out and evaluated progressively, as indicated.

**Figure 1:**
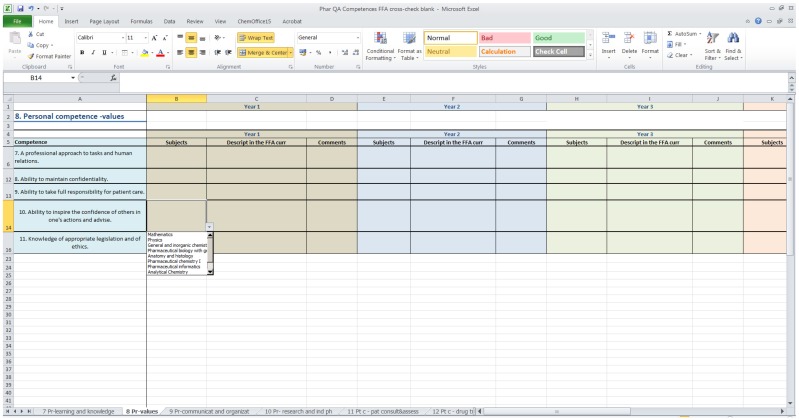
Screen-shot of the worksheet of a Microsoft Excel file generated for curricula mapping. Each worksheet includes one cluster of competences as defined by the Quality Assurance in European Pharmacy Education and Training (PHAR-QA) project (11). The competences within the clusters are listed in the ordinate. The courses in the master’s curriculum are arranged in “drop-down” form, matching the individual year of the master’s program in the abscissa.

Step 1: A Microsoft Excel file was generated composing a matrix of 50 European Pharmacy Competences Framework (EPCF) competences [13] versus 60 courses in the master’s curriculum. For greater transparency of the file, clusters are separated into individual worksheets and the competences within each cluster are listed in the *y*-axis. Courses were listed in a “drop-down” form for each year of the program in the *x*-axis (Figure 1).

Step 2: Primary mapping was done by a single member of the team, who copy-pasted the competences as described in the master’s curriculum from each course individually based on personal assessment of the matching. In cases where competences were defined more generically (covering multiple competences), they were mapped in two or more PHAR-QA competences. For example: the competence from the program “Students acquire basic knowledge about drug action within an organism and the organism’s reaction upon exposure to drug(s)” was mapped in “(29) Ability to compile and interpret a comprehensive drug history for an individual patient,” “(34) Ability to identify and prioritize drug-disease interactions (e.g., NSAIDs in heart failure) and advise on appropriate changes to medication,” and “(35) Knowledge of the bio-pharmaceutical, pharmacodynamic, and pharmacokinetic activity of a substance in the body.”

If the description was too general, such as: “Development of competences and skills of using knowledge in a particular professional area,” or not listed in the EPCF list, the faculty’s competence was listed in a separate worksheet.

Step 3: The result of the primary mapping was individually evaluated and revised by the coordinator of the master’s program, coordinator of the international student exchange, and coordinator of the traineeship. The revision was made based on their thorough knowledge of the course syllabuses.

Step 4: The final review of the mapping process and evaluation was made by all seven members of the team. Special attention was paid to:

- Competences absent from the curriculum;
- The number of times each competence was addressed in the curriculum;
- Building competences through teaching from lower to higher levels;
- Dedicated time and ECTS credits planned in the curriculum for teaching to build individual competences.

Step 5: Gaps and inconsistences in the curriculum and EPCF list were identified.

The level of agreement of scores among individual evaluators participating in the study was assessed using the Delphi methodology [14,15]. A Delphi consensus panel was run with the aim of evaluating coverage of competences as defined by the PharQA framework in the current master’s curriculum. The Delphi expert panel included four independent ratings performed by two individuals and two teams with two evaluators working together. The evaluators were six faculty professors that have insight into the pharmacy curriculum. The Delphi study consisted of two rounds. In the first round, panelists rated the coverage of the competences in the curriculum. Coverage was scored using the following five-point Likert-type scale: 0 = not covered at all, 1 = poor, 2 = fair, 3 = good, 4 = very good. Consensus on the coverage of competences was defined as the range of individual scores (Max–Min) being one or less. The panelists were also asked to provide comments on the clarity and their understanding of competences.

After the first round, the expert panel members met for a roundtable discussion. The results of the first round were presented and the panelists discussed the items for which consensus on coverage had not been attained and clarified the differences in ratings. In the second round, the panelists once again rated the coverage of competences, taking into account the roundtable discussion, the median of the panelists’ answers, and the response distribution from the first round. Consensus was defined as the range being one or less.

## 3. Results

The starting point was the EPCF list of competences, and whether and where a particular competence is present in the curriculum was checked. The Slovenian pharmacy master’s curriculum consists of 60 courses (subjects) in a 10-semester uniform program including a six-month traineeship in pharmacy, individual research work, and a master’s thesis defense. The preliminary results of the competence mapping are presented in Table 1. The numbering of the competences in the table is consistent with the numbering in the PHAR-QA project [13], in which the first six questions address the profile of the respondents (age, duration of practice, country of residence, and current occupation) and were not included in the mapping process. The questions in clusters 7–16 are reflected in 60 competences for pharmacy practice across Europe: clusters 7–10 cover personal competences, and clusters 11–17 cover patient care competences.

All competences as defined by the EPCF are covered in our master’s curriculum, although their distribution among subjects and across program years is not balanced. During the first two years of the master’s program, in which the curriculum contains typically basic subjects in the natural sciences, personal competences from clusters 7 through 11 are predominantly covered, especially those dealing with abilities to learn independently and apply logic to solve problems. Later in the program, competences from all groups are distributed more evenly. It is also evident that each subject addresses at least one EPCF competence.

The preliminary results are a rough estimate of how competences are covered in our curriculum. It was obvious that the description of competences in the curriculum was not sufficient for adequate scoring. Namely, some competences are addressed several times in a particular subject and it is not clear to what extent the competence is actually covered (i.e., mentioned, discussed, or elaborated). On the other hand, it is not possible to recognize progression in the level and sequence of student learning and performance through the program. For this reason, the evaluation was enhanced by using the Delphi approach.

Table 2 and Table 3 present coverage of competence domains and individual competences in the first and second rounds of the Delphi study. Table 4 presents consensus building between the first and second rounds of the Delphi study.

## 4. Discussion

Evaluation was performed based on the curriculum [6]. The performance of the program (i.e., educational outcomes of the competences achieved) was not part of our study. The authors of this study are aware of different approaches in curriculum mapping. The final goal is to compare intended, perceived, and achieved competences as evaluated by students, graduates, teachers, and employers. Such mapping would be very useful in improving the program and its performance [16,17]. However, for preliminary mapping with the available resources, only the first step was realistic: mapping the curriculum delivered as written in the accreditation documents, expanded by evaluation of the competences present in the curricula as explained in the section Materials and Methods.

The master’s program in pharmacy in Slovenia educates students for both aspects of pharmacy practice—working in health services and the pharmaceutical industry in approximately the same proportion—and most EPCF competences cover healthcare-oriented pharmacy practice; this is also reflected in the results of our evaluation. Personal competences are addressed with relatively higher frequencies due to the fact that the EPCF predominantly covers healthcare-oriented pharmacy competences. Namely, the definition of the pharmacy profession or pharmacy practice at the international level is not always clear [18]. There is no doubt that a pharmacist is a healthcare professional, but not only that. The pharmacist is “the university professional whose primary mission is the management and the exclusive responsibility for the formulation, preparation and the responsible dispensing of drugs to the population in addition to its inevitable participation in the protection of health and improvement of the quality of life” [19]. Several inconsistencies are evident regarding the pharmacist’s role more broadly; that is, in the pharmaceutical industry in developing and producing medicines and in laboratory medicine. The master’s program in Slovenia is designed to provide pharmacy competences within the healthcare system as well as the pharmaceutical industry, medical laboratories, research laboratories, legislation, and education. From this perspective, the PHAR-QA framework of competences does not sufficiently cover competences outside the healthcare system. Competences in drug development and production should be developed and included in greater detail.

Some definitions were found to be rather loose and/or ambiguous. For example, the competence “Knowledge of the importance of research in pharmaceutical development and practice” seems to be too general and is addressed by the majority of subjects in our curriculum. The members of the study team had difficulty understanding what the competence covers; it seems self-evident. The curriculum sets competences about research in pharmaceutical development and practice at a higher level according to Bloom’s classification [20].

It was further observed that some competences are too broad, covering multiple competences. Some examples include the following: “Ability to undertake a critical evaluation of a prescription ensuring that it is clinically appropriate and legally valid” should distinguish competences of a clinical and legislative nature/origin; “Ability to advise physicians on the appropriateness of prescribed medicines and—in some cases—to prescribe medication” should distinguish counselling (i.e., advising) from taking actions (i.e., prescribing); and “Ability to identify non-adherence to medicine therapy and make an appropriate intervention” should distinguish the ability to recognize from the ability to intervene. The problem of scoring arises when two partial competences are not from the same origin and cannot be covered in the curriculum equally. For example, prescription of medicines by a pharmacist is not allowed in many EU countries, including Slovenia. Therefore it is unreasonable to include such competences in the national curriculum.

During the education process, competences are built from lower to higher levels according to Bloom’s taxonomy: remember, understand, apply, analyze, evaluate, and create [21]. Not considering this, only courses at the top of the pillars are recognized as important for a particular competence whereas basic courses are overlooked. For example, the team had difficulty differentiating the following competences: “(7) Ability to apply current knowledge of relevant legislation and codes of pharmacy practice,” “(8) Knowledge of appropriate legislation and of ethics,” and “(9) Ability to implement general legal requirements that impact upon the practice of pharmacy”; it seems that different levels of Bloom’s classification are being addressed inconsistently. To develop competences at higher levels (i.e., to be able to perform), several lower-level competences (i.e., knowledge and skills) should be adopted and included in the curriculum. Lower-level competences are usually written very generally, such as “development of skills” or “capability of practical application of knowledge,” and are not linked to a specific field or competences. On the other hand, competence at the highest level, such as “Ability to use pharmaceutical knowledge and provide evidence-based advice on public health issues involving medicines,” means that students have already built sufficient pharmaceutical knowledge, which should be addressed inside the curriculum as separate lower-level competences (knowledge and understanding).

The roundtable discussion of the Delphi study and further analysis of the results revealed that the majority of differences in scoring arise from different perspectives on the pharmacy profession (e.g., community, hospital, industrial, academic, laboratory medicine, or regulative); for example, “7. Personal competences: learning and knowledge. 6. Ability to apply current knowledge of relevant legislation and codes of pharmacy practice.” Scoring pharmacy practice from a healthcare perspective yields different results than scoring pharmacy practice from a more general perspective, also covering industrial and regulatory aspects of the profession. Similarly, the competence “11. Patient care competences: patient consultation and assessment. 2. Ability to perform appropriate diagnostic tests, e.g., measurement of blood pressure or blood sugar” can be understood as graduates’ ability to perform some basic diagnostic tests in community pharmacy, or graduates’ ability to work in laboratory medicine (synonyms: clinical biochemistry, clinical biology) [22]. This is a common situation in Slovenia [23]. Different perspectives and understandings of competences as defined by PharQA were discussed in the roundtable, leading to more a balanced approach to evaluation among the panelists. This resulted in greater consensus in the second round of the Delphi evaluation process: the panelists reached consensus for 49 out of 50 competences.

Competences have to be designed to fit the first-day-of-job pharmacist [2,8]. From this perspective, it was found that some of the competences in the EPMF were rather too ambitious and require additional graduate training and/or specialization, as also discussed by Atkinson [13].

It can be concluded that curriculum mapping using EPMF is very useful for evaluating and recognizing weak and strong points of the curriculum. However, it must also be recognized that some additional improvement of the existing framework is needed. Namely, the competences of the framework should address various fields of the pharmacy profession in a more balanced way.

This study found the mapping process to be more complex than it seemed at the beginning. Not all of the pitfalls observed were addressed. For other mapping steps (e.g., perceived and achieved competences), some tuning differences in personal approaches would be necessary, and some kind of training would also be useful to support activities, which is in line with the recommendations of the European Commission [1] about teaching and learning improvement, and is also part of the Slovenian National Higher Education Program 2011–2020 [24].

## Figures and Tables

**Table 1:** Results of curriculum mapping of the competences in the Slovenian master’s program in pharmacy. Subjects are arranged by program years, and clusters of competences are defined by PHAR-QA. The numbers indicate how many competences from each cluster are defined in each of the subjects. Subjects are listed in alphabetical order by each year of the program.

	Cluster of Competences	7-Learning and Knowledge	8-Values	9- Communication and 9-Organisational 9-Skills	10- Research and Industrial Pharmacy	11- Patient Consultation and Assessment	12- Need for Drug Treatment	13- Drug Interactions	14- Drug dose and Formulation	15- Patient Education	16- Provision of Information and Service	17- Monitoring of Drug therapy	ECTS	Sum of All Competences Per Subject
Subject														
		Personal Competences				Patient Care Competences								
**Year 1**														
Analytical Chemistry		2	0	0	0	0	0	0	0	0	0	0	8	2
Anatomy and histology		0	0	0	0	0	1	0	0	0	0	0	4	1
General and inorganic chemistry		2	0	0	0	0	0	0	0	0	0	0	8	2
Introduction to pharmacy		1	4	0	2	0	0	0	0	0	0	0	3	7
Mathematics		1	0	0	0	0	0	0	0	0	0	0	7	1
Microbiology		0	0	0	0	1	1	0	0	0	0	0	4	2
Pharmaceutical biology with genetics		5	3	3	0	0	0	0	0	0	0	0	7	11
Pharmaceutical chemistry I		2	0	0	0	0	0	0	0	0	0	0	6	2
Pharmaceutical informatics		1	0	1	1	0	0	0	0	0	0	2	5	5
Physics		1	0	0	0	0	0	0	0	0	0	0	8	1
**Year 2**														
Organic chemistry		0	0	1	1	0	0	0	0	0	0	0	9	2
Pharmaceutical biochemistry		2	0	0	0	0	0	0	0	0	1	0	7	3
Pharmaceutical chemistry II		1	0	0	1	0	1	1	1	0	0	0	7	5
Pharmaceutical technology I		1	1	4	4	0	0	0	1	0	1	0	20	12
Physical chemistry		1	0	0	0	0	0	0	0	0	0	0	6	1
Physical pharmacy		0	0	0	1	0	0	0	0	0	0	0	5	1
Physiology		1	0	0	0	1	0	0	0	0	0	0	6	2
**Year 3**														
Cosmetology		1	0	0	1	0	0	0	0	0	0	0	5	2
Hospital Pharmacy		0	1	3	1	0	1	0	1	0	0	1	5	8
Immunology		2	0	1	0	0	1	0	0	0	0	0	5	4
Instrumental Analytical Methods in Pharmacy		1	0	0	0	0	0	0	0	0	0	0	5	1
Instrumental pharmaceutical analysis		1	0	1	1	0	0	0	0	0	0	0	4	3
Nutritional Supplements		2	2	3	0	0	0	0	0	0	1	0	5	8
Pathologic physiology		2	0	0	0	0	1	0	0	0	0	0	6	3
Pharmaceutical chemistry III		0	0	1	3	0	0	2	1	0	0	0	20	7
Pharmaceutical Marketing and Management		0	0	1	1	0	0	0	1	0	0	0	5	3
Pharmaceutical technology II		1	1	1	2	0	0	0	0	0	0	0	8	5
Pharmacoeconomics		0	0	2	0	0	0	0	0	0	0	1	5	3
Pharmacognosy I		2	2	3	0	0	0	0	0	0	1	0	9	8
Pharmacognosy II		2	2	3	1	0	0	0	1	0	0	0	4	9
Research methods in social Pharmacy		0	0	0	1	0	0	0	1	0	0	2	5	4
Social pharmacy		2	0	4	0	0	0	0	2	2	2	5	4	17
**Year 4**														
Analysis and supervision of medicinal products		2	0	0	4	0	0	0	0	0	0	0	8	6
Biochemistry of Cancer Development and Progression		1	1	0	0	0	0	0	0	0	0	0	5	2
Biopharmaceutical Evaluation of Pharmaceutical Forms		0	0	0	1	0	0	0	1	0	0	0	5	2
Biopharmaceutics with pharmacokinetics		0	0	0	2	0	1	2	2	0	0	0	9	7
Clinical chemistry		1	0	2	1	3	0	0	0	0	0	0	7	7
Clinical pharmacy		1	4	0	0	0	4	3	1	2	2	3	5	20
Design and Synthesis of Active Substances		1	0	0	1	0	0	0	1	0	0	0	5	3
Eutomers		0	0	0	2	0	0	0	1	0	0	0	5	3
Industrial pharmacy		1	0	0	4	0	0	0	0	0	0	0	5	5
Medicinal Products of alternative Medicine		3	2	2	0	0	0	0	0	0	0	0	5	7
Modified Release Pharmaceutical Forms		2	1	0	1	0	0	0	0	0	0	0	5	4
Pharmaceutical biotechnology		2	2	2	3	0	0	0	2	0	0	0	6	11
Pharmaceutical Engineering		0	0	0	1	0	0	0	0	0	0	0	5	1
Pharmacogenomics and Genetic Medicines		1	1	1	2	0	0	1	0	0	0	0	5	6
Pharmacology		1	0	0	0	0	1	2	2	0	0	0	5	6
Phytopharmaceuticals		2	2	0	0	0	0	0	0	0	1	0	5	5
Psychotropic substances and Abuse of Medicinal Products		1	0	0	0	0	2	0	0	0	0	0	5	3
Quality of Medicinal Products		0	0	0	3	0	0	0	0	0	0	0	5	3
Selected Methods of Pharmaceutical Analysis		1	0	0	0	0	0	0	0	0	0	0	5	1
Selected Topics in Clinical Biochemistry		0	0	0	0	2	0	0	0	0	0	0	5	2
Selected Topics in Pharmaceutical Biotechnology		1	2	3	2	0	0	0	1	0	0	0	5	9
Stability of medicinals		1	0	0	1	0	0	0	0	0	0	0	5	2
The Use of Genetic and Cellular Testing in Biomedicine and Pharmacy		0	1	1	0	1	0	1	0	0	0	0	5	4
Toxicological chemistry		1	0	0	1	0	1	0	0	0	0	0	5	3
**Year 5**														
Individual research work for master’s thesis		1	1	2	0	0	0	0	0	0	0	0	25	4
Master's thesis defence		2	1	2	0	0	0	0	0	0	0	0	5	5
Traineeship		3	4	7	2	1	3	3	4	3	3	3	30	36
Sum		68	39	58	57	9	18	15	25	7	13	17	410	

Legend:
1st year of study2nd year of study3rd year of study4th year of study5th year of study

**Table 2:** Coverage of competence domains as defined by the PHAR-QA framework in the Slovenian pharmacy curriculum. Results from both rounds of the Delphi study are presented as weighted medians of all competences in the domain. Coverage was scored using a five-point Likert-type scale: 0 = not covered at all, 1 = poor, 2 = fair, 3 = good, 4 = very good.

Domain	Coverage of the Competency Domain	
	1st Round Weighted Median	2nd Round Weighted Median
7. Personal competences: learning and knowledge.	3,4	3,4
8. Personal competences: values.	2,7	2,6
9. Personal competences: communication and organizational skills.	2,2	2,2
10. Personal competences: research and industrial pharmacy.	3,0	3,0
11. Patient care competences: patient consultation and assessment.	2,7	3,0
12. Patient care competences: need for drug treatment.	2,3	2,3
13. Patient care competences: drug interactions.	2,2	2,3
14. Patient care competences: drug dose and formulation.	3,3	3,2
15. Patient care competences: patient education.	2,0	2,0
16. Patient care competences: provision of information and service.	2,7	2,8
17. Patient care competences: monitoring of drug therapy.	2,0	2,0

Legend: An MS Excel three-color scale algorithm was used to present the results of the Delphi rounds, whereby the lowest value is presented in red, the highest in green, and the median in yellow.

**Table 3:** Coverage of individual competences as defined by the PHAR-QA framework in the Slovenian pharmacy curriculum. Results from both rounds of the Delphi study are presented. Coverage was scored using a five-point Likert-type scale: 0 = not covered at all, 1 = poor, 2 = fair, 3 = good, 4 = very good.

Competency Organised According to Domains	Coverage of Individual Competencies	
	1st Round Median (Min–Max)	2nd Round Median (Min–Max)
**Domain: 7. Personal competences: learning and knowledge.**		
1. Ability to identify learning needs and to learn independently (including continuous professional development (CPD).	3 (2–4)	3 (3–3)
2. Ability to apply logic to problem solving.	4 (4–4)	4 (4–4)
3. Ability to critically appraise relevant knowledge and to summarise the key points.	4 (3–4)	4 (3–4)
4. Ability to evaluate scientific data in line with current scientific and technological knowledge.	4 (3–4)	4 (4–4)
5. Ability to apply preclinical and clinical evidence-based medical science to pharmaceutical practice.	3 (2–3)	3 (3–3)
6. Ability to apply current knowledge of relevant legislation and codes of pharmacy practice.	2,5 (2–4)	2,5 (2–3)
**Domain: 8. Personal competences: values.**		
1. A professional approach to tasks and human relations.	3 (2–4)	3 (3–4)
2. Ability to maintain confidentiality.	3 (2–4)	3 (3–3)
3. Ability to take full responsibility for patient care.	2 (1–2)	2 (1–2)
4. Ability to inspire the confidence of others in one’s actions and advise.	2 (2–3)	2 (2–3)
5. Knowledge of appropriate legislation and of ethics.	3,5 (2–4)	3 (3–4)
**Domain: 9. Personal competences: communication and organisational skills.**		
1. Ability to communicate effectively—both oral and written—in the locally relevant language.	3 (3–4)	3 (3–4)
2. Ability to effectively use information technology.	2,5 (2–3)	2,5 (2–3)
3. Ability to work effectively as part of a team.	3 (2–4)	3 (3–3)
4. Ability to implement general legal requirements that impact upon the practice of pharmacy (e.g., health and safety legislation, employment law).	2,5 (2–4)	2,5 (2–3)
5. Ability to contribute to the training of staff.	1 (1–2)	1 (1–2)
6. Ability to manage risk and quality of service issues.	2 (1–2)	2 (1–2)
7. Ability to identify the need for new services.	1,5 (1–2)	1,5 (1–2)
8. Ability to understand a business environment and develop entrepreneurship.	2 (1–2)	2 (1–2)
**Domain: 10. Personal competences: research and industrial pharmacy.**		
1. Knowledge of design, synthesis, isolation, characterisation and biological evaluation of active substances.	4 (4–4)	4 (4–4)
2. Knowledge of good manufacturing practice and of good laboratory practice.	3 (3–4)	3 (3–4)
3. Knowledge of European directives on qualified persons.	1,5 (1–2)	1,5 (1–2)
4. Knowledge of drug registration, licensing and marketing.	3 (3–4)	3 (3–4)
5. Knowledge of the importance of research in pharmaceutical development and practice.	3,5 (2–4)	3,5 (3–4)
**Domain: 11. Patient care competences: patient consultation and assessment.**		
1. Ability to interpret basic medical laboratory tests.	4 (1–4)	4 (3–4)
2. Ability to perform appropriate diagnostic tests e.g., measurement of blood pressure or blood sugar.	1 (0–3)	2 (0–3)
3. Ability to recognise when referral to another member of the healthcare team is needed.	3 (2–3)	3 (2–3)
**Domain: 12. Patient care competences: need for drug treatment.**		
1. Ability to retrieve and interpret information on the patient’s clinical background.	3 (1–3)	3 (3–3)
2. Ability to compile and interpret a comprehensive drug history for an individual patient.	2 (1–3)	2 (2–3)
3. Ability to identify non-adherence to medicine therapy and make an appropriate intervention.	2 (1–3)	2 (2–2)
4. Ability to advise to physicians on the appropriateness of prescribed medicines and—in some cases—to prescribe medication.	2 (1–3)	2 (1–2)
**Domain: 13. Patient care competences: drug interactions.**		
1. Ability to identify and prioritise drug-drug interactions and advise appropriate changes to medication.	3 (2–3)	3 (2–3)
2. Ability to identify and prioritise drug-patient interactions, including those that prevent or require the use of a specific drug, based on pharmaco-genetics, and advise on appropriate changes to medication.	1,5 (1–3)	2 (1–2)
3. Ability to identify and prioritise drug-disease interactions (e.g., NSAIDs in heart failure) and advise on appropriate changes to medication.	2 (1–2)	2 (1–2)
**Domain: 14. Patient care competences: drug dose and formulation.**		
1. Knowledge of the bio-pharmaceutical, pharmacodynamic and pharmacokinetic activity of a substance in the body.	4 (4–4)	4 (4–4)
2. Ability to recommend interchangeability of drugs based on in-depth understanding and knowledge of bioequivalence, bio-similarity and therapeutic equivalence of drugs.	3 (2–4)	3 (3–4)
3. Ability to undertake a critical evaluation of a prescription ensuring that it is clinically appropriate and legally valid.	2,5 (1–3)	2 (2–2)
4. Knowledge of the supply chain of medicines thus ensuring timely flow of quality drug products to the patient.	3 (2–3)	3 (2–3)
5. Ability to manufacture medicinal products that are not commercially available.	4 (3–4)	4 (3–4)
**Domain: 15. Patient care competences: patient education.**		
1. Ability to promote public health in collaboration with other professionals within the healthcare system.	2 (2–3)	2 (2–3)
2. Ability to provide appropriate lifestyle advice to improve patient outcomes (e.g., advice on smoking, obesity, etc.).	2 (2–3)	2 (2–3)
3. Ability to use pharmaceutical knowledge and provide evidence-based advice on public health issues involving medicines.	2 (2–3)	2 (2–3)
**Domain: 16. Patient care competences: provision of information and service.**		
1. Ability to use effective consultations to identify the patient’s need for information.	2 (1–3)	2 (1–2)
2. Ability to provide accurate and appropriate information on prescription medicines.	3,5 (3–4)	3,5 (3–4)
3. Ability to provide evidence-based support for patients in selection and use of non-prescription medicines.	2,5 (2–4)	3 (3–4)
**Domain: 17. Patient care competences: monitoring of drug therapy.**		
1. Ability to identify and prioritise problems in the management of medicines in a timely and effective manner and so ensure patient safety.	2 (2–3)	2 (2–3)
2. Ability to monitor and report Adverse Drug Events and Adverse Drug Reactions (ADEs and ADRs) to all concerned, in a timely manner, and in accordance with current regulatory guidelines on Good Pharmacovigilance Practices (GVPs).	1,5 (1–2)	1,5 (1–2)
3. Ability to undertake a critical evaluation of prescribed medicines to confirm that current clinical guidelines are appropriately applied.	2,5 (2–3)	2,5 (2–3)
4. Ability to monitor patient care outcomes to optimise treatment in collaboration with the prescriber.	2 (1–2)	2 (1–2)
5. Ability to contribute to the cost effectiveness of treatment by collection and analysis of data on medicines use.	2 (1–3)	2 (2–3)

Legend: Results from the second round of the Delphi study that are shaded represent medians that changed from the first round of the Delphi study.

**Table 4:** Consensus building between the first and second rounds of the Delphi study. The frequency of ranges of individual scores (Max–Min) evaluating coverage of individual competences as defined by the PHAR-QA framework in the Slovenian pharmacy curriculum.

Range of Individual Scores (Max–Min)		2nd Round					
		0	1	2	3	4	Sum
**1st Round**	**0**	3	0	0	0	0	**3**
	**1**	2	25	0	0	0	**27**
	**2**	6	12	0	0	0	**18**
	**3**	0	1	0	1	0	**2**
	**4**	0	0	0	0	0	**0**
	**Sum**	**11**	**38**	**0**	**1**	**0**	**50**
